# Physical Activity in Patients with Celiac Disease: A Systematic Review

**DOI:** 10.3390/nu18142400

**Published:** 2026-07-22

**Authors:** Irene Zapata-Martínez, Marta Herrador-López, Víctor Manuel Navas-López, Lara Bossini-Castillo, Teresa Nestares, Rafael Martín-Masot

**Affiliations:** 1 Department of Pharmacology and Pediatrics, School of Medicine, University of Málaga, 29071 Málaga, Spain; irenezapata365@uma.es (I.Z.-M.); rafammgr@gmail.com (R.M.-M.); 2 Biomedical Research Institute of Málaga (IBIMA), University of Málaga, 29590 Málaga, Spain; herradorlopezm@gmail.com; 3 Pediatric Gastroenterology and Nutrition Unit, Hospital Regional Universitario de Málaga, 29010 Málaga, Spain; victor.navas@gmail.com; 4 Department of Genetics, Institute of Biotechnology, Centre for Biomedical Research (CIBM), University of Granada, 18071 Granada, Spain; 5 Human Reproduction and Hereditary and Complex Diseases (IBS-TEC14), Advanced Therapies and Biomedical Technologies, Institute of Biomedical Research of Granada, ibs.GRANADA, 18012 Granada, Spain; 6 Department of Physiology, Faculty of Pharmacy, University of Granada, 18071 Granada, Spain; nestares@ugr.es; 7 Institute of Nutrition and Food Technology “José Mataix Verdú” (INYTA), Biomedical Research Centre (CIBM), University of Granada, 18071 Granada, Spain

**Keywords:** celiac disease, physical activity, quality of life, gluten-free diet, systematic review

## Abstract

**Objectives**: Celiac disease (CD) is an immune-mediated disorder triggered by gluten ingestion. Although a gluten-free diet (GFD) is the only effective treatment, patients may still experience inflammation, nutritional imbalances, and reduced quality of life. Physical activity (PA) has demonstrated anti-inflammatory benefits; however, its role in CD management remains unclear. This systematic review aimed to synthesize evidence on the associations of PA and the outcomes of exercise interventions in individuals with CD, focusing on metabolic, nutritional, clinical, and functional outcomes. **Methods**: Following PRISMA guidelines, we included peer-reviewed observational and experimental studies assessing PA in individuals with CD across all age groups, without language or date restrictions. Searches were conducted in PubMed, Scopus, Web of Science, EMBASE, and SPORTDiscus. The risk of bias was evaluated using JBI and Cochrane RoB 2 tools. **Results**: Fourteen studies (17 publications) were included, the vast majority of which were cross-sectional, along with one quasi-experimental study and two randomized controlled trial cohorts reported across five publications. Data from these predominantly observational studies suggested possible, yet inconsistent, links between higher PA levels and better profiles in body composition, inflammatory and oxidative markers, quality of life, and psychological outcomes. Positive associations were also observed in some studies regarding gastrointestinal symptoms and adherence to the GFD. However, findings on metabolic markers and bone mineral density were inconsistent and linked to dietary factors. **Conclusions**: While PA represents a potential adjunct in the comprehensive management of CD, particularly in relation to functional and inflammatory outcomes. However, the current evidence remains highly preliminary, limited, and inconsistent, which restricts the strength of any definitive conclusions. Therefore, high-quality, longitudinal studies and well-designed clinical trials are needed to confirm its long-term benefits, especially in the pediatric population, and to establish specific recommendations.

## 1. Introduction

Celiac disease (CD) is a complex, multifactorial systemic condition triggered by the ingestion of gluten and its prolamins, which affects genetically susceptible individuals and is characterized by a severe immune-mediated inflammatory reaction [1].

The only effective treatment currently available is a strict gluten-free diet (GFD). A late diagnosis or failure to diagnose CD can lead to continued gluten exposure, which triggers sustained chronic inflammation. The exacerbated immune response associated with this situation could lead to an increased risk of developing other diseases, including malignant tumors. Some studies have reported a higher incidence of tumors the later the diagnosis is made and in patients who do not follow a GFD [2,3,4]. A link has been demonstrated between chronic inflammation in CD and the development of lymphoproliferative syndromes, such as T-cell lymphoma [5,6]. A strict GFD appears to have immunomodulatory effects, as evidenced by a reduction in the expression of genes associated with inflammation, along with the restoration of immune homeostasis through regulatory immune mechanisms [7,8].

The GFD must meet the recommended nutritional targets for energy and nutrients, just as it does for the general population. However, the reality is that it is usually a restrictive and unbalanced diet, which replaces naturally gluten-containing foods with their ultra-processed gluten-free equivalents. Our studies in the celiac population, as well as those by other authors, on long-term adherence to the GFD show that energy and nutrient intake fall far short of recommendations, with excessive consumption of fats, proteins and simple carbohydrates and insufficient intake of fiber and complex carbohydrates, as well as certain vitamins and minerals, possibly due to the exclusion of naturally fiber-rich cereals and the inclusion of commercial (ultra-processed) gluten-free products, which contain higher levels of refined flours and saturated fats than their gluten-containing counterparts [5,9,10,11,12,13,14,15]. Several studies have linked the consumption of ultra-processed food (UPF) with the development of chronic inflammatory diseases, including CD itself [16,17,18]. Diets based on processed and UPF could cause intestinal dysbiosis (i.e., excessive growth of opportunistic microorganisms or pathogenic species) which may trigger a pro-inflammatory immune response, increased intestinal permeability and susceptibility to autoimmune diseases, such as CD [19]; indeed, one of the first steps in the pathophysiology of CD is the disruption of the tight junctions in the small intestinal epithelium, increasing its permeability [20]. This could explain the persistently elevated levels of circulating cytokines (e.g., IL-4, IL-10, IL-1, IL-8 and IL-21) found in adult celiac patients [21] and in children [22], even after long-term adherence to a GFD. In this context, although data on the pediatric population is limited, children with CD who consume fewer UPFs have better inflammatory markers and oxidative status [9,23].

Furthermore, the management of CD poses two distinct, major implications for patients’ health-related quality of life (QoL) depending on their dietary behavior. On one hand, maintaining strict lifelong adherence to a GFD requires significant lifestyle adjustments and substantial social restrictions [24]. Patients frequently experience social isolation, anxiety during social gatherings due to a persistent fear of cross-contamination, and the constant psychological burden of explaining their condition to others [24] to the extent that they may experience fatigue and a reduction in social activities and levels of physical activity (PA) [25]. On the other hand, non-compliance or accidental gluten exposure leads to severe clinical manifestations driven by autoimmune-mediated malabsorption. This path can result in chronic gastrointestinal distress, nutritional deficiencies (such as anemia and osteoporosis), and long-term systemic complications, including refractory sprue or malignancy. Psychologically, non-adherence often generates a cyclic pattern of distress, guilt, and a severely degraded health perception [24]. Consequently, both absolute adherence and non-compliance represent distinct clinical and psychosocial pathways that profoundly undermine the QoL in individuals with CD, creating a critical need for complementary therapeutic strategies such as PA. PA is a recognized strategy for reducing the risk of chronic diseases, and recent research has focused on its role in improving the inflammatory profile, with large population-based cohort studies having consistently shown an inverse association between markers of systemic inflammation and PA [26].

PA is associated with improvements in traditional health parameters (weight, blood pressure, glucose), but it also appears to be related to modulations in the immune system and chronic inflammation, which could be key in conditions such as diabetes, obesity, cardiovascular disease, and other inflammatory disorders [27], including CD. Lifestyle interventions, including PA, may help regulate the redox state and reduce inflammation in children [28]. However, further studies are needed to refine our understanding of the mechanisms linking PA to low-grade systemic inflammation, the magnitude of this relationship, and the amount of PA required to promote clinically significant changes in the pathological link between inflammation and disease.

Despite the well-established benefits of PA in chronic conditions with inflammatory and autoimmune components, such as obesity, diabetes, and cardiovascular disease, its specific role in managing CD is unclear. Although PA could represent a useful complementary strategy to improve inflammatory, metabolic, nutritional and clinical outcomes in this population, the available evidence is still limited and has not been synthesized comprehensively. In this context, the aim of this systematic review (SR) was to evaluate the scientific evidence on the association of PA with metabolic-nutritional, clinical, and functional variables in CD.

## 2. Materials and Methods

### 2.1. Study Design

This SR was conducted in accordance with the PRISMA guidelines (Preferred Reporting Items for Systematic Reviews and Meta-Analyses) [29], and the study objectives were defined using the PICO/PECO framework (Population, Intervention/Exposure, Comparator, and Outcomes) (Table 1). To improve transparency and reproducibility, the review protocol and supplementary methodological materials have been made publicly available on the Open Science Framework (OSF) (https://osf.io/k68a4/).

### 2.2. Eligibility Criteria

Given the limited availability of randomized controlled trials (RCTs) in the CD population, this SR included both experimental and observational study designs. Only articles published in peer-reviewed journals were considered, with no restrictions on year or language of publication. When full-text articles were unavailable in English or Spanish, automated translation tools were used to assess eligibility. Regarding participants, the study includes human subjects of any age with CD while excluding animal or in vitro studies. In terms of article type, it includes peer-reviewed journal articles with full-text availability, but excludes systematic or narrative reviews, meta-analyses, clinical practice guidelines, case reports, editorials, letters to the editor, study protocols, comments or opinions, and conference abstracts or proceedings. The study design includes observational studies (e.g., prospective cohort, cross-sectional, and case–control studies) and RCTs, but excludes non-human experimental studies.

### 2.3. Search Strategy

Five tailored search strategies were developed using a combination of MeSH terms and free-text keywords related to CD or the GFD, and PA. MeSH terms were applied in PubMed, whereas equivalent controlled vocabulary terms and keywords were used in the remaining databases. These strategies were applied to PubMed, Scopus, Web of Science, EMBASE, and SPORTDiscus. The complete search equations used for each database are provided in the Supplementary Materials (File S1).

The search conducted in Web of Science was limited to the Web of Science Core Collection, as it integrates the subject categories Sport Sciences, Nutrition & Dietetics, Gastroenterology & Hepatology, and other fields relevant to the scope of this review. The Web of Science search was limited to the Web of Science Core Collection because it integrates subject categories such as Sport Sciences, Nutrition & Dietetics, and Gastroenterology & Hepatology, which are relevant to this review’s scope.

A total of 144, 346, 277, 351, and 56 records were retrieved from PubMed, Scopus, Web of Science, EMBASE, and SPORTDiscus, respectively. The final searches were conducted on 24 July 2025.

### 2.4. Study Selection

Two investigators (IZ and RM) screened the titles and abstracts of all retrieved records independently against the predefined inclusion and exclusion criteria. Potentially relevant articles were then evaluated in full text. During both stages of the selection protocol, any disagreements between the primary reviewers were resolved through discussion until a consensus was reached; in cases where a disagreement persisted, a third investigator (TN) made the final decision. The entire screening and selection process, along with the reasons for excluding studies at the full-text stage, was documented and tracked using a standardized spreadsheet.

### 2.5. Data Extraction, Outcomes, and Data Synthesis

**Figure 1:**
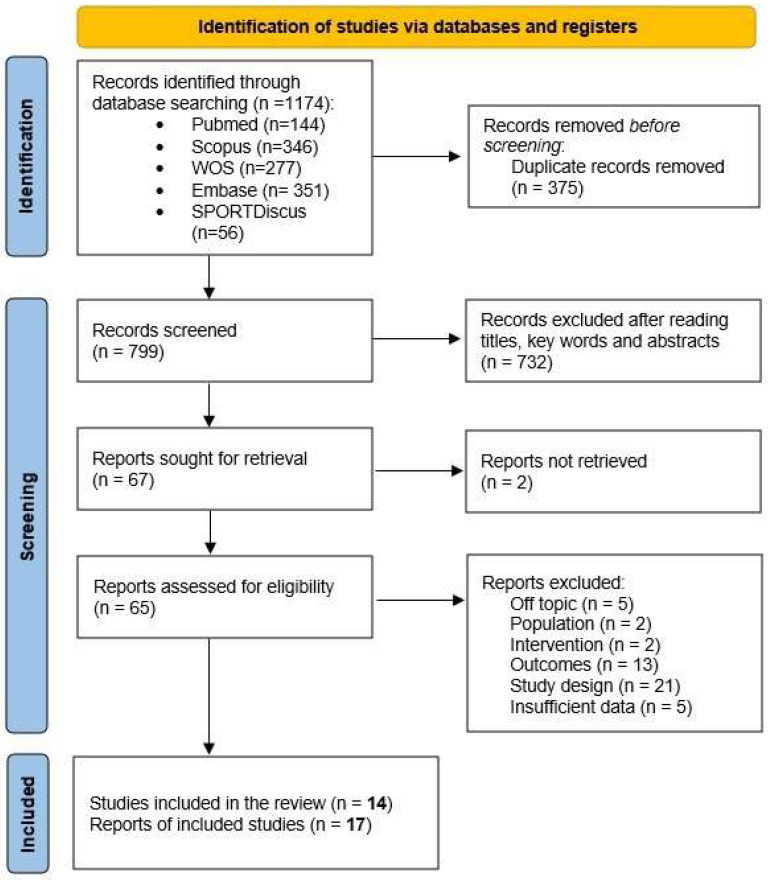
PRISMA flow diagram.

As shown in the PRISMA flow diagram [30], in Figure 1, a total of 1.174 records were identified after the initial search. After removing 375 duplicate records using the Mendeley application, 799 records were screened, of which 732 were excluded for not meeting the inclusion criteria. Subsequently, the full texts of 65 out of 67 articles were retrieved and assessed for eligibility through full-text review. The reasons for excluding each full-text article were recorded.

Finally, 14 distinct studies were included in the SR. Some of these studies yielded multiple publications, such as secondary analyses or follow-up papers. Thus, these 14 studies corresponded to a total of 17 separate reports, all of which are cross-referenced.

### 2.6. Validity Assessment

The risk of bias of the included studies was assessed according to study design. For RCTs, the Cochrane Risk of Bias 2 (RoB 2; 2019 version) tool was used to assess the risk of bias [31]. The JBI Critical Appraisal Tools were applied to observational studies: the JBI Cohort Checklist for cohort studies, the JBI Quasi-Experimental Checklist for quasi-experimental studies, and the JBI Analytical Cross-Sectional Checklist for cross-sectional studies, following the methodological guidance provided in the JBI Manual for Evidence Synthesis [32,33].

Two researchers (IZ and RM) conducted the risk of bias assessment independently. Any disagreements between the lead reviewers were resolved through discussion until a consensus was reached; in cases where disagreement persisted, a third researcher (TN) made the final decision.

### 2.7. Data Management and Synthesis

A standardized data extraction template was developed in Microsoft Excel to ensure consistency across studies. Data were extracted and synthesized to summarize the findings in accordance with the review’s objectives. Any discrepancies between reviewers were resolved through discussion and consensus.

A formal assessment of the feasibility of a meta-analysis was performed. However, a quantitative statistical synthesis was precluded due to severe clinical and methodological heterogeneity across the included studies. Specifically, the narrative synthesis was deemed the only appropriate approach due to: (1) substantial differences in the populations studied, ranging from pediatric cohorts to postmenopausal adult populations; (2) a lack of standardization in the instruments used to assess PA, which included self-reported questionnaires (e.g., IPAQ) to non-validated indices and structured resistance training protocols; and (3) highly divergent outcome measures, spanning heterogeneous biochemical, nutritional, and functional clinical variables. Consequently, pooling the data was mathematically and methodologically inappropriate. Furthermore, due to the exploratory scope of this review, the substantial heterogeneity of outcomes and PA measures, and the absence of a quantitative synthesis, a formal GRADE assessment was not performed. 

## 3. Results

A total of 14 studies were included in the SR, corresponding to 17 published reports. Of the 14 included studies, 11 were cross-sectional studies, one was a quasi-experimental study, and two were randomized controlled trials. These two trials were reported in five publications (three by Martínez-Rodríguez et al. and two from the Canadian MOVE-C trial).

To facilitate a structured analysis of the role of PA in CD, the evaluated endpoints were classified into three distinct categories based on standard clinical-epidemiological frameworks. Metabolic–nutritional outcomes include objective biochemical, inflammatory, microbiota, and anthropometric metrics. The clinical outcomes included direct manifestations of the disease, organic complications, and clinically relevant comorbidities associated with CD, including somatic and psychiatric dimensions. Lastly, functional outcomes include patient-reported measures that reflect how the disease impacts daily functioning, psychological well-being, and behavior. These measures include health-related QoL, mood alterations, self-compassion, and dietary adherence patterns. This categorization ensures a clear distinction between organic-somatic markers and multidimensional functional/well-being indicators (Table 2).

The main characteristics of the included studies, study design, study population, study objectives, assessment methods, key findings, and conclusions, are summarized in Table 3 (Cross-sectional studies) and Table 4 (Intervention studies: RCTs and Quasi-Experimental Studies).

### Risk of Bias Assessment

**Figure 2:**
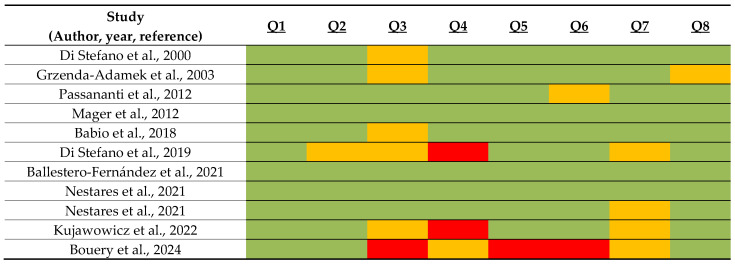
Quality assessment of cross-sectional studies using the JBI Critical Appraisal Checklist for Analytical Cross-Sectional Studies. Q1: Inclusion criteria clearly defined; Q2: Study subjects and setting described; Q3: Exposure measured validly and reliably; Q4: Objective criteria used for outcome measurement; Q5: Confounding factors identified; Q6: Strategies to deal with confounding factors stated; Q7: Outcomes measured validly and reliably; Q8: Appropriate statistical analysis used. Color coding: green: Yes (low risk of bias); yellow: unclear/partial (moderate risk of bias); red: No (high risk of bias); blue: not applicable. Cited studies: [9,15,38,39,40,41,42,43,45,46,48].

Among the cross-sectional studies, assessed using the JBI Critical Appraisal Checklist for Analytical Cross-Sectional Studies, most presented one or no domains with a moderate risk of bias (Figure 2). The study by Di Stefano et al. [42] showed a moderate risk of bias in domains Q2, Q3, and Q7, and a high risk in domain Q4. Kujawowicz et al.’s study [46] presented a moderate risk of bias in domains Q3 and Q7 and a high risk in domain Q4. Finally, the study by Bouery et al. [48] showed a moderate risk of bias in domains Q4 and Q7 as well as a high risk of bias in three domains.

The quasi-experimental study by Costa et al. [34] showed a low risk of bias across all domains according to the JBI Critical Appraisal Checklist for Quasi-Experimental Studies (see Supplementary Table (File S2)).

**Figure 3:**
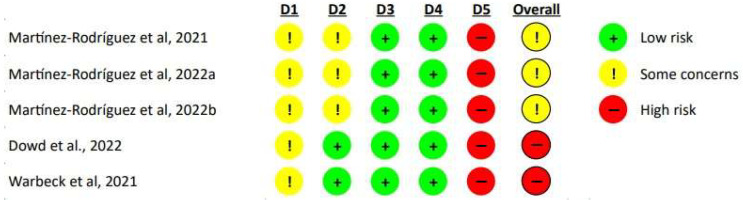
Quality assessment of randomized controlled trials using the Cochrane Risk of Bias 2 tool. D1: Bias arising from the randomization process; D2: Bias due to deviations from intended interventions; D3: Bias due to missing outcome data; D4: Bias in measurement of the outcome; D5: Bias in selection of the reported result. Cited studies: [35,36,37,44,47].

Finally, regarding the RCT evidence, assessment using the Cochrane Risk of Bias 2 tool Regarding the RCT evidence, the Cochrane Risk of Bias 2 tool revealed that the three publications by Martínez-Rodríguez et al. [35,37,44], derived from the same randomized clinical trial, were deemed to have some concerns regarding the overall risk of bias. Likewise, the MOVE-C trial, reported in two publications by Warbeck et al. [36] and Dowd et al. [47], was deemed to have a high overall risk of bias, mainly due to some concerns in domain D1 (randomization process) and a high risk of bias in domain D5 (selection of the reported results) (Figure 3).

## 4. Discussion

To ensure consistency and clarity of presentation, the discussion has been organized according to the same domains categorized in the results, distinguishing between metabolic-nutritional, clinical, and functional outcomes.

**(a)**  **Metabolic-nutritional outcomes:**

The results suggest that PA is associated with a better inflammatory status in CD beyond that achieved with a GFD [9,34]. Importantly, these associations are supported by studies with generally low methodological risk of bias. This chronic inflammation is central to the pathophysiology of CD, which is a complex process that is not yet fully understood. Exposure to gluten triggers an abnormal innate and adaptive immune response that generates autoantibodies capable of affecting not only the intestine but also other tissues. In this context, oxidative stress plays a significant role, as gliadin induces an increase in reactive oxygen species (ROS) and the release of pro-inflammatory cytokines such as interleukin-1 (IL-1), interleukin-15 (IL-15), and tumor necrosis factor (TNF) [49,50].

Nestares et al. (2021) [9] observed a sustained inflammatory state in children with CD following a GFD, suggesting that additional mechanisms underlie the persistence of inflammation even in the absence of direct gluten exposure. In this regard, a higher intake of UPF and lower levels of PA are linked to a more pronounced pro-inflammatory profile [9], supporting the promotion of PA as a complementary strategy to the GFD [25].

These findings have also been reported in adult populations with CD. Costa et al. [34] observed a reduction in C-reactive protein (CRP) and IL-6 in a quasi-experimental study of a group that combined fish oil supplementation with aerobic PA, demonstrating a more favorable anti-inflammatory profile than that observed obtained with supplementation alone.

Consistently, higher levels of PA are associated with lower levels of inflammatory markers, including cytokines and CRP [51,52]. Conversely, higher levels of inflammatory mediators have been reported in patients with CD who do not respond to a GFD, particularly in those with a higher symptom burden [53]. Taken together, these results support the role of PA as a promising adjunctive intervention to optimize the inflammatory state in CD, in line with findings in other chronic diseases with an inflammatory component, such as ischemic heart disease, type 2 diabetes, or metabolic syndrome [54,55].

Another relevant finding was the relationship between PA and the gut microbiota. Several studies have shown that moderate exercise promotes a healthy immune system and increases the diversity of the gut microbiome [56]. In this context, patients with CD have been reported to exhibit gut dysbiosis [57], characterized by a predominance of pro-inflammatory groups, which may persist even after diagnosis and adherence to a GFD [58].

Regarding PA and the microbiota in patients with CD, the MOVE-C trial showed that a 12-week high-intensity interval training (HIIT) program produced potentially beneficial changes in the beta diversity of the microbiota [36]. Moreover, these findings should be interpreted with caution, as the trial presented a high risk of bias in the selection of the reported results. However, since these modifications were not maintained during the follow-up period, the long-term clinical relevance of exercise on the gut microbiome remains uncertain. Consequently, these findings must be interpreted with caution and treated as strictly preliminary and non-conclusive at this stage.

Furthermore, celiac patients who follow a GFD may be at an increased risk of being overweight or developing metabolic syndrome [59]. In this context, several studies included in this review have analyzed the relationship between PA and metabolic syndrome markers in the celiac population. The MOVE-C trial observed no significant changes in cholesterol or glucose levels, blood pressure, waist circumference, or body mass index (BMI) following a 12-week structured PA intervention, although a decrease in resting heart rate was recorded, suggesting early improvements in cardiovascular fitness [36]. Other studies in adults with CD have reported favorable outcomes, including reductions in fat mass, weight, and BMI [34,37], while in the pediatric population, PA has been associated with higher lean body mass [43].

Overall, the evidence suggests that PA is associated with more favorable inflammation, microbiota, and cardiometabolic profile in CD patients. However, further studies are needed to confirm these associations and establish clear recommendations.

**(b)**  **Clinical Outcomes:**

The risk of reduced bone mineral density (BMD) in people with CD is well documented [60,61]. This condition can affect growth in children [62] and increase the risk of osteoporosis and fractures in adults. Consequently, clinical guidelines recommend paying specific attention to bone health in celiac patients of all ages [63,64].

Most of the studies included in this review have analyzed the relationship between PA and bone quality. Regarding this relationship, PA was only positively associated with higher BMD in only two cross-sectional studies, both of which were assessed as having a low risk of bias according to the JBI Critical Appraisal Checklist. In untreated adult celiac patients, PA was independently associated with femoral BMD, though not lumbar BMD [38]. Similarly, in the pediatric population, a trend toward a positive association was observed between vigorous PA and a higher BMD/Z-score, although bone health showed a more consistent relationship with adherence to the Mediterranean diet [43].

Conversely, other studies found no significant associations between PA and BMD in children, adolescents, and adults [39,40,41,42,44]. These findings should be interpreted with caution, as some of these studies had methodological limitations, including issues related to PA assessment, outcome measurement, confounding factors, or selective reporting, which may have contributed to the inconsistency of the available evidence. These results suggest that the relationship between PA and BMD may be modulated by other factors. In these studies, bone quality was primarily associated with other variables, notably adherence to the GFD, which was consistently identified as a key determinant of BMD [65].

In the pediatric population, nutritional factors such as calcium, protein, and vitamin K intake have been found to be significantly associated with BMD [39,40]. In adults with CD, deficiencies in calcium and vitamin D, as well as other micronutrients, have also been reported, particularly in the context of a long-term GFD [15], which may relate to BMD alongside factors such as BMI and persistent inflammation [38,42]. This could explain, at least in part, the lack of a consistent association between PA and bone health observed in some studies.

Regarding bone outcomes, the synthesized literature indicates that PA does not have a primary, direct relationship with higher BMD in patients with CD. In fact, no significant exercise-outcome interactions were found across most evaluated studies. Instead, strict compliance with a GFD and adequate nutritional factors (such as calcium, vitamin D, protein intake, and overall diet quality) emerge as the primary and most consistent determinants of bone health in this population. PA appears to play a secondary, supportive role rather than acting as a major independent correlate of bone recovery.

Another important aspect of CD is the increased risk of developing eating disorders (EDs). A recent meta-analysis has described a bidirectional association between CD and EDs [66]. Since PA can be used as a weight management strategy [67], it is necessary to evaluate its role in this population. However, a RCT found no changes in eating attitudes following a combined GFD and PA intervention in adult women with CD [35].

These findings should be interpreted with caution, as the trial showed some concerns about the overall risk of bias, mainly due to a high risk of bias in selecting the reported results. In adolescents with CD, moderate PA was associated with higher scores on screening scales for EDs (SCOFF scale) and bulimia nervosa (BITE scale), although no consistent relationships were found with the other instruments used [45].

These findings suggest that the relationship between PA and the risk of EDs in CD is inconclusive and may be linked to multiple factors. Due to multifactorial nature of EDs, as well as their higher prevalence in adolescents [68] and in the CD population, screening tools should be incorporated to detect them early in celiac patients [66]. At the same time, further prospective studies with large samples in adolescent populations are needed to establish definitive conclusions regarding the relationship between PA and the risk of EDs in CD.

In addition to EDs, depression and anxiety are among the psychiatric disorders associated with CD [69]. The likelihood of these disorders is significantly higher in the CD population than in the non-CD population (OR of 6.03 and 2.17, respectively), according to the meta-analysis by Clappison et al. [70]. This association has also been described in other chronic diseases, such as diabetes mellitus, cardiovascular diseases, COPD, and cancer, among others [71].

In the case of CD, although the underlying causes are not fully understood, but both biological and social factors have been proposed. Among the biological factors, immunological mechanisms related to chronic inflammation, nutritional deficiencies resulting from intestinal malabsorption, and possible direct effects on the central nervous system stand out [72]. In this context, the pain and chronic inflammation associated with gastrointestinal disorders could affect the anterior cingulate cortex, promoting neuroinflammatory processes that increase vulnerability to anxiety and depression and disrupting the regulation of the autonomic and immune nervous systems [73].

Furthermore, social factors also play a significant role. Adherence to GFD has been associated with a reduction in anxiety, but with persistent depressive symptoms in some patients [74].

Additionally, a GFD can have negative social consequences, including isolation and avoidance of social situations due to fear of cross-contamination, or the constant need to explain the condition to others [70,75,76]. In this context, PA has a positive relationship with psychological well-being. Various studies have demonstrated PA’s effectiveness in alleviating depression and anxiety symptoms in patients with chronic conditions, including chronic pain, HIV or kidney disease. These studies have shown a significantly lower severity of these disorders and more favorable scores depending on the type and intensity of the exercise [77,78]. These positive outcomes have been linked to lower psychological stress, the release of neurotransmitters associated with well-being, and better general physical fitness, which may assist patients in coping better with their illness [79].

Additionally, PA levels also appear to be associated with certain clinical manifestations, such as fewer digestive symptoms [47] and less severe menopause-related symptoms in women with CD [44]. However, these findings should be interpreted with caution, as both RCTs had concerns about the randomization process and a high risk of bias related to the selection of the reported results. Beyond these clinical outcomes, PA may also be related to broader functional and psychosocial outcomes in CD, as discussed in the following section.

**(c)**  **Functional outcomes:**

Beyond its associations with clinical manifestations and psychiatric comorbidities, PA may also be related to broader functional and psychosocial dimensions of CD. Consistent with this idea, the results of this review indicate that PA in patients with CD is associated with positive outcomes such as higher levels of self-compassion [47], better mood [44], and no overall increase in fatigue [41]. In addition, physically active patients showed a presented a lower prevalence of orthorexia risk in one study [46]. However, these associations should be interpreted with caution, because some supporting studies had methodological limitations, particularly regarding selective reporting of results, objective outcome measurement, and, to a lesser extent, the randomization process and control of potential confounding factors.

Reduced QoL has been reported in patients with CD. Kurppa et al.’s review [80] notes that most studies show a poorer QoL in untreated CD patients compared with healthy individuals. Patients adhering to a GFD typically exhibit better QoL scores than untreated individuals, though they often do not reach normal levels. These results suggest that although diet remains the cornerstone of treatment, persistent limitations may remain linked to patients’ overall well-being.

In this regard, PA has been linked to a higher overall QoL in numerous chronic conditions, and is associated with significantly better indicators of perceived general health and vitality [81].

In the context of CD, the observed association between PA and a higher disease-specific QoL [47], together with the positive associations described when combined with the GFD [37], provides evidence of PA’s potential role as a complementary strategy in the comprehensive management of the disease. These findings support the view that PA should be considered a relevant component within the comprehensive management of CD, with the potential to optimize both QoL and certain clinical parameters.

Another important aspect from a behavioral perspective, another important aspect is the potential interaction between PA and treatment adherence. Engaging in PA has been observed to be associated with better adherence to the GFD than following the diet alone [35], as well as being linked to fewer gastrointestinal symptoms [47]. In turn, greater adherence to the GFD appears to be associated with higher levels of PA and a better QoL [48], suggesting a possible bidirectional relationship between dietary compliance and adopting an active lifestyle. However, caution is warranted when interpreting these observations, because the supporting studies had methodological limitations, particularly related to selective reporting of results, exposure measurement, and the identification and management of potential confounding factors. Overall, the available evidence suggests that PA constitutes a relevant strategy for improving QoL, psychological well-being, and treatment-related behaviors in people with CD.

**(d)**  **Methodological Considerations and Interpretation of the Findings:**

Importantly, extreme caution is required when interpreting the synthesized evidence due to the studies’ methodological limitations and significant clinical heterogeneity. According to the JBI and Cochrane RoB 2 assessments, some of the included studies presented methodological limitations that may have influenced the reported findings. Among the randomized controlled trials, the main concerns were related to the selection of the reported results and, to a lesser extent, the randomization process. In contrast, some observational studies showed limitations in the objective measurement of outcomes and the management of potential confounding factors. The diversity of the cohorts reviewed in this systematic study further compounds this vulnerability. The cohorts span from pediatric populations to postmenopausal women and include highly variable durations of adherence to a GFD and a lack of standardization in PA assessment tools.

Consequently, these limitations preclude broad and sweeping generalizations. While the positive shifts observed across the metabolic, clinical, and functional domains are highly promising, they cannot be considered definitive yet. Instead, these health benefits must be interpreted strictly as potential trends within specific sub-populations, emphasizing that subsequent stratified confirmation through high-quality, standardized research is required before universal clinical recommendations can be formulated.

## 5. Strengths and Limitations

The key strengths of this review include the comprehensive and rigorous search strategy, which included peer-reviewed articles with no restrictions on language or year of publication, and consultation of five leading international health sciences and PA databases. The review was conducted in accordance with the PRISMA guidelines, ensuring transparency and methodological rigor, and employed validated tools for the assessment of risk of bias. Additionally, categorizing the results allows for a more systematic and consistent presentation. Furthermore, validated tools were used to assess the risk of bias.

This review has several limitations. First, a major methodological limitation of the current body of evidence relies heavily on observational, cross-sectional designs, which inherently preclude establishing causal relationships. While these studies provide valuable insights into the lifestyles of patients with CD, they only capture associations, meaning that positive health parameters cannot be definitively attributed to PA levels alone. Furthermore, robustness of the available evidence about exercise interventions is significantly limited by the small number of independent clinical trial cohorts available in the literature. Specifically, multiple published papers evaluating physical and metabolic responses (such as those by Martínez-Rodríguez et al.) originate from the exact same underlying trial population. Dependence on identical patient cohorts restricts the diversity of intervention evidence because the observed benefits reflect the characteristics and compliance of a single study sample rather than widely reproducible clinical findings across different geographic or demographic CD groups. Consequently, the high reliance on cross-sectional data, paired with a small number of independent trial populations and generally modest sample sizes, substantially lowers the overall strength of the conclusions.

Furthermore, the included studies showed high heterogeneity in terms of design, sample size, population characteristics and methodologies used, hindering the comparison and generalization of the results. This variability encompasses differences in age, sex, time since diagnosis, adherence to the GFD and PA level, as well as a lack of research in the pediatric population. Furthermore, most studies assessed general or self-reported PA, but did not specifically analyze structured PA programs, which limit the interpretation of their relations. Finally, the lack of standardization in measuring PA, which is often based on self-reported instruments that are not always validated, may affect the accuracy and reproducibility of the findings. These findings underscore the necessity of longitudinal studies and well-designed clinical trials to elucidate the role of PA in the comprehensive management of CD.

## 6. Conclusions

In conclusion, the available, largely observational literature provides preliminary evidence that PA is positively associated with higher QoL and lower reporting of psychological symptoms in patients with CD who adhere to a GFD. In specific cohorts, PA exhibited potential trends toward better emotional well-being, greater self-compassion, lower gastrointestinal symptoms, and higher adherence to a GFD. However, due to the high clinical and methodological heterogeneity (particularly regarding age ranges, small sample sizes, and diverse exercise assessment tools), these observations should be strictly interpreted as potential trends rather than definitive outcomes. Evidence remains conflicting regarding metabolic outcomes and BMD. Given that the current landscape of evidence is limited and inconsistent, future highly standardized, multicenter RCTs are essential to validate these preliminary trends and provide stratified, evidence-based exercise guidelines for this population.

## Figures and Tables

**Table 1:** PICO/PECO framework for study selection.

Variable	Definition
Population	Patients of any age with Celiac Disease
Interventions/Exposure	Engagement in physical activity
Comparators	No comparator required
Outcomes	Favorable profiles or changes in clinical status, metabolic or nutritional parameters, and/or functional outcomes

**Table 2:** Classification of included studies based on Physical Activity outcome categories.

Outcome Category	Specific Outcomes Evaluated	Reference(s)
Metabolic–nutritional	Inflammatory profile	[9,34]
	Anthropometric changes	[15,34,35,36,37]
	Nutritional status	[15]
	Gut microbiota composition	[36]
Clinical	Bone mineral density	[38,39,40,41,42,43,44]
	Risk of eating disorders and orthorexia	[35,45,46]
	Symptom improvements (digestive and menopause-related)	[44,47]
	Resting heart rate	[36]
Functional	Adherence to a gluten-free diet and its interaction with physical activity	[35,48]
	Quality of life and self-compassion	[37,47,48]
	Improvements in mood	[44]

**Table 3:** Cross-sectional studies included.

Author (Year, Country)	Study Design and Population	Study Objective and Relationship with Physical Activity	Outcomes and Assessment Methods	Main Results and Conclusions
Di Stefano et al., 2000 (Italy) [38]	Cross-sectional analytical; *N* = 39 untreated CD (18–55 y); 74% women; 18 symptomatic, 21 subclinical.	To assess clinical and lifestyle factors associated with BMD. PA was examined in relation to BMD (clinical outcomes).	PA: Non-validated composite index (occupational activity, active transport, leisure/sport, and activities of daily living) in which a higher score indicates a greater level of PA, self-reported. Primary outcome: lumbar and femoral BMD (DEXA, Z-score). Other outcomes: BMI, clinical severity, smoking, sun exposure, sex, and albumin.	PA is higher in subclinical vs. symptomatic (6.8 ± 1.5 vs. 4.25 ± 0.9; *p* < 0.02). PA positively correlated with BMD (lumbar r = 0.57; femoral r = 0.71). ANCOVA: PA was independently associated with femoral BMD only (*p* = 0.011). Clinical severity and BMI: main factors associated with BMD; sex: lumbar BMD. Conclusion: BMD is primarily associated with clinical severity and BMI; higher PA levels are associated with greater femoral BMD, and sex predicts lumbar BMD.
Grzenda-Adamek et al., 2003 (Poland) [39]	Cross-sectional analytical; *N* = 59 CD adolescents (10–20 y); 68% girls; groups by GFD adherence.	To evaluate the relationship between GFD adherence on BMD and physical development in children and adolescents with CD. PA was examined in relation to BMD (clinical outcomes).	PA: habitual PA (non-validated questionnaire), low (<2 h/week) vs. high (>7 h/week). Primary outcome: lumbar BMD L2–L4 (DEXA, Z-score) Other outcomes: adherence to GFD, calcium intake, height/weight.	PA not associated with BMD (NS). BMD was significantly lower in poor GFD adherence vs. strict and occasional (*p* = 0.01 and *p* = 0.003, respectively). High Ca intake is associated with higher BMD (*p* = 0.002). Growth unaffected. Conclusion: BMD is mainly associated with GFD adherence and calcium intake, not PA.
Mager et al., 2012 (Canada) [40]	Cross-sectional analytical; *N* = 43 children with newly diagnosed CD (3–17 y); *N* = 33 at 1-year.	To examine the relationship between vitamins D and K and bone mineral status at diagnosis and after 1 year of GFD. PA was examined in relation to BMD and BMC (clinical outcomes).	PA: age-validated questionnaires (Adolescent Physical Activity Recall / FELS). Primary outcomes: BMD and BMC (whole body and lumbar spine; DEXA). Other outcomes: anthropometry, Tanner stage, vitamin D, PTH, Ca/P/Mg, vitamin K (PIVKA-II), bone turnover markers (BAP, total osteocalcin, NTX), 3-day dietary record, sun exposure (validated questionnaire).	PA and sun exposure were not associated with BMD, BMC, or bone turnover markers (NS); PA levels remained unchanged over 1 year. Vitamin K deficiency (23%) normalized after 1 year (*p* < 0.05), and vitamin D insufficiency decreased from 43% to 25% (*p* < 0.05). Higher protein and vitamin K intake were associated with higher BMC (*p* < 0.001 and *p* = 0.003, respectively). Conclusion: Nutrition showed a stronger association with bone health than PA during the first year after diagnosis.
Passananti et al., 2012 (Italy) [41]	Cross-sectional with prospective follow-up; *N* = 110 women with newly diagnosed CD (20–60 years). Group 1: 2-year GFD follow-up (*N* = 55); Group 2: 5-year GFD follow-up (*N* = 55).	To evaluate the relationship between PA, fatigue, and BMD in women with CD. PA was examined in relation to BMD (clinical outcomes) and fatigue (functional outcomes).	PA: IPAQ short form at diagnosis and after 2 or 5 years of GFD. Primary outcomes: BMD (lumbar spine and femur; DEXA, WHO criteria) and fatigue (VAS). Other outcomes: weight, height, BMI, age at menarche, menopausal status, nutritional markers, vitamin D, anti-tTG antibodies, calcium intake (dietitian-assessed), smoking, alcohol consumption, gastrointestinal symptoms, pharmacological treatment, GFD adherence (VAS), and lifestyle factors.	PA levels at follow-up: 2-year GFD: 53.2% low PA; 10.6% high PA. 5-year GFD: 46.3% low PA; 14.6% high PA. PA levels unchanged over time (NS). No association between PA and BMD at diagnosis or follow-up; no association between ΔPA and ΔBMD (NS). No relationship between PA and fatigue, except an isolated finding of higher fatigue in women with high PA at 5-year GFD follow-up (*p* = 0.039). BMD increased after 2 and 5 years of GFD. Conclusion: PA shows a weaker association with bone health compared to compliance with a GFD.
Babio et al., 2018 (Spain) [45]	Cross-sectional case–control study (COEDATTI); *N* = 196 (98 CD, 98 healthy controls); 10–23 y.	To assess the risk of eating disorders in individuals with CD compared with healthy controls. PA was examined in relation to the risk of eating disorders (clinical outcomes).	PA: self-reported leisure-time PA (IOM categories: sedentary, moderate, active). Primary outcomes: eating disorder risk (ChEAT, EAT-26, SCOFF, BITE, BSQ, Figure Drawings Scale). Other outcomes: BMI, sociodemographic questionnaire, dietary intake (24-h recall).	PA distribution: 26% sedentary in CD vs. 16% in controls; 40% moderate PA in CD vs. 49% in controls (NS). CD patients > 13 years of age had higher EAT-26 scores than controls (β = 2.15; *p* = 0.04). No clear differences in ED risk using other screening tests. Conclusion: CD was associated with higher eating attitude scores in adolescents, but not with higher overall ED risk; Lower PA levels in CD compared with controls (NS). Cross-sectional design limits causal inference.
Di Stefano et al., 2019 (Italy) [42]	Cross-sectional study; *N* = 44 adult CD patients on long-term strict GFD (mean time since CD diagnosis: 11 years). Groups: (a) normal BMD, (b) low BMD.	To evaluate local and systemic factors associated with bone loss in CD patients on long-term strict GFD. PA was examined in relation to BMD loss (clinical outcomes).	PA: self-reported PA and sun exposure questionnaire (referenced from previous literature; non-validated; PA/exercise not clearly differentiated). Primary outcomes: biochemical markers of bone metabolism (OPG, RANKL, OPG/RANKL ratio). Other outcomes: BMI.	No differences in PA levels between normal and low BMD groups (NS). Patients with low BMD showed higher serum OPG and higher OPG/RANKL ratio, with similar RANKL levels. Conclusion: persistent inflammation may contribute to bone loss in long-term treated CD, reflecting impaired bone matrix production. PA was not associated with BMD loss.
Ballestero- Fernández et al., 2021 (Spain) [15]	Cross-sectional study; *N* = 148 adults: 64 CD adults on long-term GFD (>1 year) and 74 non-celiac controls; mean age 39 y.	To evaluate nutritional status through dietary intake, body composition, biochemical parameters, and PA in adults with CD on GFD. PA was examined in relation to nutritional status (metabolic–nutritional outcomes).	PA: IPAQ questionnaire. Primary outcomes: dietary intake (24 h dietary records). Other outcomes: BMI, fat mass %, BMD, blood parameters (folate, 25-OH vitamin D, PTH, iron).	No significant differences in PA between celiac and non-celiac adults; most participants reported moderate to vigorous PA. CD adults showed insufficient intake of folates, vitamin E, iodine, calcium, and iron (women), with very low vitamin D intake, and 34% showing moderate vitamin D deficiency. Both groups followed high-fat, high-protein, low-carbohydrate diets. CD women appeared more prone to osteopenia/osteoporosis. Conclusion: micronutrient status should be routinely monitored in CD adults; habitual PA associated with better bone health markers despite inadequate calcium and vitamin D intake.
Nestares et al., 2021 (Spain) [43]	Cross-sectional study; *N* = 99 children: CD (*N* = 59; long >18 months or recent <18 months GFD) and non-celiac (*N* = 40); mean age 10–11 y.	To assess the relationship between MD adherence and PA on body composition and bone health in children with CD. PA was examined in relation to body composition and BMD (clinical outcomes)	PA: PAQ. WHO PA guideline compliance: ≥60 min/day of moderate-to-vigorous PA. Primary outcomes: BMD and body composition (lean, fat, and bone mass; DEXA); Mediterranean Diet adherence (KIDMED). Other outcomes: BMI, clinical, and sociodemographic data.	CD children showed lower body weight, lean mass, BMC, and bone Z-score vs. controls, regardless of GFD duration. Greater time in vigorous PA was associated with higher lean mass (*p* = 0.020) and higher BMD/Z-score (trend, *p* = 0.078). Higher MD adherence was associated with higher bone Z-score (*p* = 0.020). Conclusion: MD adherence and vigorous PA are associated with better bone health and body composition in children with CD.
Nestares et al., 2021 (Spain) [9]	Cross-sectional study; *N* = 85 children: CD (*N* = 53; long > 18 months or recent <18 months GFD) and healthy controls (*N* = 32); mean age ~10 y; 69.8% girls (CD).	To assess the association of ultra-processed food (UPF) consumption on oxidative stress and inflammatory profile in children with CD. PA was examined in relation to oxidative and inflammatory biomarkers (metabolic–nutritional outcomes).	PA: PAQ questionnaire. Primary outcomes: oxidative/antioxidant status (SOD, isoprostanes, TAS) and inflammatory markers (cytokine panel); UPF consumption (24 h recall, NOVA). Other outcomes: BMI, clinical and sociodemographic data, and MD adherence (KIDMED).	Children with CD showed higher UPF consumption than controls (*p* < 0.05). Lower UPF intake (<50% daily energy) was associated with higher Mediterranean Diet adherence, higher moderate PA levels, and a healthier oxidative and inflammatory profile (*p* < 0.05), independent of GFD duration. Conclusion: High UPF consumption combined with lower PA and poor MD adherence is associated with a worse inflammatory and oxidative profile; PA promotion should accompany dietary interventions in children with CD.
Kujawowicz et al., 2022 (Poland) [46]	Cross-sectional study; *N* = 123 CD females, mean age 34 ± 8.7 years, BMI 21.4 kg/m^2^.	To examine the prevalence of orthorexia risk in individuals with CD. PA was examined in relation to orthorexia risk (clinical outcomes).	PA: self-reported PA and frequency (non-validated questionnaire). Primary outcomes: risk of orthorexia (ORTO-15 questionnaire). Other outcomes: eating habits (dietary survey), sociodemographic.	70% of those at risk for orthorexia and 81% of those without risk did some PA. Individuals with risk for orthorexia were significantly less likely to run (17%) and to exercise aerobically (31%) than those without the risk of orthorexia. Conclusion: The prevalence of orthorexia in CD cannot be clearly established; PA was lower in individuals at risk.
Bouery et al., 2024 (Lebanon) [48]	Cross-sectional study; *N* = 136 CD patients (78% female, 18–59 y).	To assess the relationship between GFD and the social quality of life (QoL) and PA PA examined in relation to QoL and GFD adherence (functional outcomes).	PA: self-reported PA and frequency (adapted IPAQ short form questionnaire). Primary outcomes: GFD adherence and its association with QoL (validated questionnaire). Other outcomes: sociodemographic, BMI.	Significant association between CD following GFD and higher PA levels (*p* = 0.0001). Conclusion: GFD is positively associated with PA levels, contributing to a better overall QoL.

Abbreviations: ANCOVA, analysis of covariance; anti-tTG, anti-tissue transglutaminase antibodies; BAP, bone alkaline phosphatase; BITE, Bulimia Investigatory Test of Edinburgh; BMI, body mass index; BMC, bone mineral content; BMD, bone mineral density; BSQ, Body Shape Questionnaire; Ca, calcium; CD, celiac disease; ChEAT, Children’s Eating Attitudes Test; COEDATTI, Cohort of Eating Disorders and Attitudes in Teenagers; DEXA, dual-energy X-ray absorptiometry; EAT-26, Eating Attitudes Test–26; GFD, gluten-free diet; HR, heart rate; IOM, Institute of Medicine; IPAQ, International Physical Activity Questionnaire; ISAK, International Society for the Advancement of Kinanthropometry; KIDMED, Mediterranean Diet Quality Index for children and adolescents; MD, Mediterranean diet; Mg, magnesium; NOVA, NOVA food classification system; NS, not significant; NTX, *N*-terminal telopeptide of type I collagen; OPG, osteoprotegerin; ORTO-15, Orthorexia Nervosa Questionnaire–15 items; PA, physical activity; PAQ, Physical Activity Questionnaire; PIVKA-II, protein induced by vitamin K absence or antagonism-II; PTH, parathyroid hormone; QoL, quality of life; RANKL, receptor activator of nuclear factor κB ligand; SCOFF, Sick, Control, One stone, Fat, Food questionnaire; SOD, superoxide dismutase; TAS, total antioxidant status; UPF, ultra-processed foods; VAS, visual analogue scale; WHO, World Health Organization.

**Table 4:** Intervention studies: Randomized Controlled Trials and Quasi-Experimental Studies.

Author (Year, Country)	Study Design and Population	Intervention and Relationship with Physical Activity	Outcomes and Assessment Methods	Main Results and Conclusions
**Martínez-Rodríguez** **et al.,** **2021** (Spain) [37]	RCT; *N* = 28 postmenopausal women with CD. **Groups:** GFD + PA (*N* = 7), GFD alone (*N* = 7), CD control (*N* = 7), non-CD control (*N* = 7).	Supervised **PA (PA)** program (ACSM-based), 3–4×/wk, 12 wk + **GFD** PA intervention was examined in relation to **quality of life (QoL) and muscle strength (functional outcomes) and anthropometric changes (metabolic–nutritional outcomes).**	**Primary outcome:** QoL (QoL; WHOQOL-BREF). **Other outcomes:** body composition (bioelectrical impedance), handgrip strength (dynamometry).	Only GFD + PA **↑ QoL** (psychological and environmental domains), **↓ fat mass** (−4.8%, *p* < 0.001), **↑ fat-free mass** (+7.2%, *p* < 0.01), and **↑ handgrip strength** (+15%, *p* < 0.05) vs. GFD alone/controls. **Conclusion:** PA adds clear functional and body-composition benefits to GFD.
**Warbeck** **et al.,** **2021** (Canada) [36]	RCT; *N* = 41 inactive adults with CD. **Groups:** High-intensity interval training (HIIT) + lifestyle education (*N* = 20), waitlist control group (*N* = 21).	High-intensity interval training (HIIT) 2×/wk, 12 wk + lifestyle education PA intervention was examined in relation to **gut microbiota, MetS markers, and anthropometric changes (metabolic–nutritional outcomes) and heart rate (HR) (clinical outcomes).**	**Primary outcome:** feasibility and HR. **Other outcomes:** gut microbiota (16S rRNA sequencing), metabolic syndrome biomarkers (blood), blood pressure, waist circumference.	**Gut microbiota:** No significant changes in α-diversity; significant effect on β-diversity (*p* = 0.037) after HIIT+. Most taxa changes did not persist at 3-month follow-up, except HIIT+ showed enrichment of *Burkholderiaceae*. **Metabolic syndrome biomarkers:** No significant effects on cholesterol, glucose, blood pressure, waist circumference, or BMI. **Resting heart rate**: decreased following the intervention. **Conclusion:** HIIT and lifestyle education are feasible in CD patients and show beneficial shifts in microbiota composition and reduced resting HR.
**Martínez-Rodríguez** **et al.,** **2022** (Spain) [35]	RCT; *N* = 28 postmenopausal women with CD. **Groups:** GFD + PA (*N* = 7), GFD alone (*N* = 7), CD control (*N* = 7), non-CD control (*N* = 7).	Supervised elastic-band resistance training, 3×/wk for 12 wk, with progressive intensity monitored by Borg scale. PA intervention was examined in relation to **GFD adherence and risk of eating disorders (clinical outcomes) and anthropometry (metabolic–nutritional outcomes).**	**Primary:** adherence to GFD (Celiac Dietary Adherence Test). **Other outcomes:** eating attitudes (EAT-26), blood markers, anthropometry (ISAK).	GFD + PA group: **↑ adherence to GFD** (+30%, *p* < 0.001); **no changes** in eating attitudes, blood markers or anthropometry. **Conclusion:** PA improves adherence to the GFD without adverse effects on eating attitudes.
**Martínez-Rodríguez et al., 2022** (Spain) [44]	RCT; *N* = 28 postmenopausal women with CD. **Groups:** GFD + PA (*N* = 7), GFD alone (*N* = 7), CD control (*N* = 7), non-CD control (*N* = 7).	Supervised elastic-band resistance training, 3×/wk for 12 wk, with progressive intensity monitored by Borg scale. PA intervention was examined in relation to **menopausal symptoms (urogenital symptoms and vigor) and bone quality (clinical outcomes) and mood (functional outcomes).**	**Primary outcome:** menopausal symptoms (Menopause Rating Scale). **Other outcomes:** mood (Profile of Mood States (POMS-29)), bone quality (calcaneal ultrasound).	GFD + PA **↓ urogenital symptoms** (*p* < 0.01) and **↑ vigor** (POMS subscale) (*p* < 0.001); **no change** in bone quality. **Conclusion:** Combined GFD and resistance exercise improved menopausal symptoms and mood but did not modify bone quality over 12 weeks.
**Dowd et al., 2022** (Canada) [47]	RCT; *N* = 41 inactive adults with CD.	HIIT plus lifestyle education, 2×/week for 12 weeks, with progressive high-intensity intervals (~90% HRmax) monitored by heart rate vs. waitlist group. PA intervention was examined in relation to **QoL, gastrointestinal symptoms, and self-compassion (functional and clinical outcomes).**	**Primary outcome:** CD-specific quality of life (CD-QoL). **Other outcomes:** GFD adherence (CDAT), gastrointestinal symptoms (CeD-GSRS), self-compassion (questionnaire), sleep quality (questionnaire).	Post-intervention **↑ QoL** (+23%, *p* < 0.05), **↓ GI symptoms** (−15%, *p* < 0.01), ↑ **self**-**compassion** (EMM = 3.42, SE = 0.14, *p* = 0.005), ↑ **GFD adherence** (EMM = 10.22; SE = 0.43; *p* = 0.04); improvements in **gastrointestinal symptoms** (EMM = 1.54; SE = 0.14; *p* = 0.02). Some gains were not maintained at the 3-month follow-up. **Conclusion**: Inactive adults with CD improved CD-specific QoL and PA levels, with effects maintained at 3 months. GFD adherence, gastrointestinal symptoms, and self-compassion also improved post-intervention but were not sustained at follow-up.
**Costa** **et al.** **2022** (Brazil) [34]	**Quasi-experimental (non-randomized);** *N* = 31 adults (19 CD, 12 healthy); 3 parallel groups: (1) Fish oil supplementation (2 g/day EPA + DHA), (2) Aerobic PA + supplementation (3×/wk, 12 wk, 60–70% HRmax), (3) Healthy control (no intervention).	Aerobic PA (3×/wk, 12 wk, 60–70% HRmax) + fish oil (2 g/day EPA + DHA) vs. fish oil alone vs. control. PA intervention was examined in relation to **inflammatory markers and anthropometric changes (metabolic–nutritional outcomes).**	**Primary outcome:** inflammatory markers (C-reactive protein (CRP), interleukin-6; blood). **Other outcomes:** anthropometry (weight, BMI, body composition), lipid profile (blood), dietary intake (3-day record).	↓ CRP (−25%, *p* < 0.01) and ↓ IL-6 (−50%, *p* = 0.03) with PA+ supplementation; supplementation alone ↓ IL-6 but not CRP; no lipid changes. ↓ Weight and BMI in the PA group (*p* = 0.0422 and 0.0418, respectively). **Conclusion**: Aerobic PA provides anti-inflammatory benefits beyond supplementation alone.

**Abbreviations**: ACSM, American College of Sports Medicine; BMI, body mass index; CD, celiac disease; CD-QoL, Celiac Disease Quality of Life questionnaire; CDAT, Celiac Dietary Adherence Test; CeD-GSRS, Celiac Disease Gastrointestinal Symptom Rating Scale; CRP, C-reactive protein; EAT-26, Eating Attitudes Test–26; EMM, estimated marginal means; EPA, eicosapentaenoic acid; GFD, gluten-free diet; HIIT, high-intensity interval training; HR, heart rate; HRmax, maximum heart rate; IL-6, interleukin-6; ISAK, International Society for the Advancement of Kinanthropometry; MetS, metabolic syndrome; n, sample size; PA, physical activity; POMS-29, Profile of Mood States–29 items; QoL, quality of life; RCT, randomized controlled trial; SE, standard error; WHOQOL-BREF, World Health Organization Quality of Life–BREF version; ↑, increase; ↓, decrease. **Note**: Martínez-Rodríguez et al. [35,37,44], and Warbeck et al. [36] and Dowd et al. [47] represent multiple publications derived from the same underlying randomized trials. These publications were retained separately in the table because they reported different outcomes but were counted as single studies in the overall study count.

## Data Availability

The original contributions presented in the study are included in the article/Supplementary Materials; further inquiries can be directed to the corresponding author.

## Supplementary Materials

The following supporting information can be downloaded at https://www.mdpi.com/article/10.3390/nu18142400/s1: Table S1. Search strings used. Table S2. Quality assessment of non-randomized intervention studies using the JBI Critical Appraisal Checklist for Quasi-Experimental Studies.
