# Supplementary material for: Physical Activity in Patients with Celiac Disease: A Systematic Review
Source: Nutrients. 2026 Jul 22;18(14):2400. doi: 10.3390/nu18142400 (PMC13415151; doi:10.3390/nu18142400)
Supplement: Supplementary file 1 [file nutrients-18-02400-s001.zip › nutrients-4369034-supplementary.pdf]

SUPPLEMENTARY MATERIAL S1

Table S1. Search strings used

Database	Search strategy	Notes
**Pub Med**	(Celiac Disease[Mesh] OR "celiac disease"[tiab] OR "coeliac disease"[tiab] OR "Gluten Enteropathy"[tiab]) AND (Exercise[Mesh] OR Exercise Therapy[Mesh] OR Physical Fitness[Mesh] OR Motor Activity[Mesh] OR Sports[Mesh] OR Physical Exertion[Mesh] OR "physical activity"[tiab] OR "exercise"[tiab] OR "fitness"[tiab] OR "sport"[tiab] OR "physical exertion"[tiab])	The systematic search in PubMed was conducted by combining MeSH terms and keywords in the title/abstract. Search date: 21 July 2025.
**Scopus**	(TITLE-ABS-KEY ("celiac disease" OR "coeliac disease" OR "gluten enteropathy")) AND (TITLE-ABS-KEY ("physical activ*" OR exercis* OR "exercise therapy" OR "physical fitness" OR "motor activity" OR sport* OR "physical exertion" OR fitness))	Search date: 22 July 2025.
**Web of Science**	TS= (celiac* OR coeliac* OR "gluten enteropathy") AND TS= ("physical activity" OR exercise OR "exercise therapy" OR "physical fitness" OR "motor activity" OR sport* OR "physical exertion" OR fitness)	The search in Web of Science was performed within the Web of Science Core Collection, as it integrates the categories *Sport Sciences*, *Nutrition & Dietetics*, *Gastroenterology & Hepatology*, and other fields relevant to the topic of the review.<br>Search date: 22 July 2025.
**EMBASE**	#1 'celiac disease'/exp OR 'celiac disease':ti,ab OR 'coeliac disease':ti,ab OR 'gluten enteropathy':ti,ab<br><br>#2 'exercise'/exp OR 'exercise therapy'/exp OR 'physical fitness'/exp OR 'motor activity'/exp OR 'sport'/exp OR 'physical exertion'/exp OR 'physical activity':ti,ab OR exercise:ti,ab OR fitness:ti,ab OR sport:ti,ab OR 'physical exertion':ti,ab<br><br>#3 #1 AND #2<br><br>#4 #3 AND ('Article'/it OR 'Article in Press'/it OR 'Clinical Trial'/it)	Search date: 23 July 2025.

**SPORTDiscus**	(TI "celiac disease" OR AB "celiac disease" OR SU "celiac disease" OR TI "coeliac disease" OR AB "coeliac disease" OR SU "coeliac disease" OR TI "gluten enteropathy" OR AB "gluten enteropathy" OR SU "gluten enteropathy") AND (TI exercise OR AB exercise OR SU exercise OR TI "physical activity" OR AB "physical activity" OR SU "physical activity" OR TI fitness OR AB fitness OR SU fitness OR TI sport* OR AB sport* OR SU sport* OR TI "physical exertion" OR AB "physical exertion" OR SU "physical exertion")	The search in SPORTDiscus was conducted via the EBSCOhost platform.<br>Search date: 24 July 2025.

SUPPLEMENTARY MATERIAL S2

Table S2. Quality assessment of non-randomized intervention studies using the JBI Critical Appraisal Checklist for Quasi-Experimental Studies**.**

Author (year)	Q1	Q2	Q3	Q4	Q5	Q6	Q7	Q8	Q9
Costa & Brito, 2022									

Note 1: Q1: Clear identification of cause and effect; Q2: Similarity of participants in comparison groups; Q3: Similar treatment or care across groups other than the intervention of interest; Q4: Presence of a control group; Q5: Multiple measurements of outcomes pre- and post-intervention; Q6: Completeness of follow-up and appropriate handling of losses; Q7: Reliability of outcome measurement; Q8: Consistency of outcome measurement across groups or over time; Q9: Appropriate statistical analysis. **Note 2:** Colour coding: Green: Yes (low risk of bias); Yellow: Partial/unclear (moderate risk of bias); Red: No (high risk of bias); Blue: Not applicable.
